# Is body weight dissatisfaction a predictor of depression independent of body mass index, sex and age? Results of a cross-sectional study

**DOI:** 10.1186/s12889-016-3497-8

**Published:** 2016-08-24

**Authors:** Aline Richard, Sabine Rohrmann, Tina Lohse, Monika Eichholzer

**Affiliations:** Division of Chronic Disease Epidemiology, Epidemiology, Biostatistics and Prevention Institute, University of Zurich, Hirschengraben 84, CH-8001 Zurich, Switzerland

**Keywords:** Depression, Body weight, Weight dissatisfaction, Switzerland

## Abstract

**Background:**

Little is known about the association of dissatisfaction with body weight - a component of body image - with depression in individuals of different sex, age, and with different body mass index (BMI). Hence, the aim of our study was to evaluate the association of body weight dissatisfaction (BWD) with depression in different sub-groups.

**Methods:**

We analyzed data of 15,975 individuals from the cross-sectional 2012 Swiss Health Survey. Participants were asked about their body weight satisfaction. The validated Patient Health Questionnaire (PHQ-9) was used to ascertain depression. Age was stratified into three groups (18–29, 30–59, and ≥60 years). The body mass index (BMI) was calculated from self-reported body height and weight and categorized into underweight (BMI <18.5 kg/m^2^), normal weight (BMI 18.5–24.9 kg/m^2^), overweight (BMI 25.0–29.9 kg/m^2^), and obesity (BMI ≥30 kg/m^2^). The association between body weight dissatisfaction (BWD) and depression was assessed with logistic regression analyses and odds ratios (OR) with 95 % confidence intervals (CI) were computed.

**Results:**

BWD was associated with depression in the overall group (OR 2.04, 95 % CI 1.66–2.50) as well as in men (OR 1.85, 95 % CI 1.34–2.56) and women (OR 2.25, 95 % CI 1.71–2.96) independent of BMI. The stratification by age groups showed significant associations of BWD with depression in young (OR 1.78, 95 % CI 1.16–2.74), middle-aged (OR 2.10, 95 % CI 1.61–2.74) and old individuals (OR 2.34, 95 % CI 1.30–4.23) independent of BMI. Stratification by BMI categories resulted in statistically significant positive associations of BWD and depression in underweight, normal weight, overweight and obese individuals.

**Conclusion:**

BWD was associated with depression independent of BMI, sex and age.

## Background

Body image is a complex concept that embraces numerous components including feelings, attitudes, perceptions, and behaviors toward one’s body [1, 2]. In Western countries, research concentrated on the body’s appearance, especially on body shape and body weight [3], which is a component of body image [4]. Body image [5] and body weight dissatisfaction (BWD) [6] are very common in adolescents and adults. A German study showed that 48 % of 25–74 year old women and 33.2 % of men of the same age were affected by body weight dissatisfaction [6].

An increasing number of studies indicate the importance of a healthy body image, particularly in association with eating disorders [7], but also in the context of mental health and depression [8]. Depression is an important public health problem worldwide [9]. According to the World Health Organization, it accounts for 4.3 % of the global burden of disease and is an important cause of disability worldwide (11 % of all years lived with disability) [10]. Thus, it is of great importance to reveal risk and protective factors for this multifactorial disease.

Research on the association between various components of body image and depression by some authors has focused on adolescents [11, 12]. In a 4-year prospective study among female adolescents, body dissatisfaction, dietary restraint, and bulimic symptoms at study entry predicted the development of major depression among initially not depressed individuals [13]. Body dissatisfaction was significantly related to depression in the bivariate but not in the multivariate analysis. The evidence for an association between various components of body image and depression of female adolescents is based on a number of studies [14, 15], whereas the literature is rather limited for males [12].

In addition, much less is known about this association in adult males and females of different age groups [8, 16, 17]. Not all sub-groups of a population may have the same psychological functioning [11, 18]. Furthermore, the association between body image and BWD and depression may not only depend on sex and age, but also on one’s own body weight [19].

Obese individuals are frequently exposed to stigma, which can lead to poor psychological outcomes [20], such as lower self-esteem and depression [12]. A recent systematic review and meta-analysis revealed a bi-directional relationship between depression and obesity among adult men and women [21]. In the present study, we aimed to evaluate the association of BWD with depression overall, in men and women, as well as in young, middle-aged and old individuals independent of body weight. We used data of the 2012 Swiss Health Survey, a population-based representative sample of the adult population living in Switzerland.

## Methods

### Participants and data

We used data from the Swiss Health Survey (SHS) 2012, which was conducted by the Swiss Federal Statistical Office (SFSO) (Legal basis: Ordinance of the Conduct of Federal Statistical Surveys of 20 June 1993). The SHS does not require formal approval by an ethics committee. It is a population-based cross-sectional study, aiming to collect information about health status, several lifestyle and demographic factors, and healthcare use over time. The SHS was carried out every 5 years since 1992. Participants were selected based on registries of inhabitants using a stratified random sampling technique. In the SHS 2012, a total of 21,597 individuals aged 15 years or older and living in a private household participated. This corresponds to a response rate of 54 %. Besides a computer-assisted telephone interview, a written questionnaire was provided (paper or online) upon approval from the participants (*n* = 18,357). The multistage probability sampling and the appropriate weighting factors provided by the SFSO ensures the representativeness of the Swiss population.

Data on depression was obtained from the written questionnaire and 16,980 individuals provided information on depression status. After excluding adolescents (<18 years; *n* = 16,349), pregnant women (*n* = 130) as well as individuals with missing information on body mass index (BMI) (*n* = 90) and on body weight satisfaction (*n* = 20) our sample consisted of 16,109 individuals. In a further step, all individuals with missing information on confounders were excluded, resulting in a final sample of 15,975 individuals.

### Measurements

BWD was assessed by one question in the telephone interview asking if one is satisfied with his/her body weight. Answers were categorized into “absolutely satisfied”, “rather satisfied”, “rather unsatisfied”, and “absolutely unsatisfied”. We then dichotomized these answers into “satisfied” and “unsatisfied” with body weight.

Depression status was considered as the outcome of our analysis. In the 2012 SHS, depression was assessed in the written questionnaire with the Patient Health Questionnaire (PHQ-9). This short screening questionnaire is a valid tool to assess depression by scoring on each of the 9 DSM-IV criteria for major depressive episodes and is broadly used in both practice and research [22, 23]. From a possible total score of 27 the cut-off point of ≥ 10 has shown a sensitivity of 88 % and a specificity of 88 % for the diagnosis of current major depression [22, 23]. Thus, we dichotomized a participant's PHQ-9 score into < 10 (no depression) and ≥ 10 (depression).

Potential confounders in our analyses were self-reported weight and height. BMI was calculated as weight in kg divided by squared height in meter and was categorized into underweight (BMI < 18.5 kg/m^2^), normal weight (BMI 18.5–24.9 kg/m^2^), overweight (BMI 25.0–29.9 kg/m^2^), and obesity (BMI ≥ 30 kg/m^2^). Additional self-reported data of the participants were obtained on sex, age, area of residence (urban, rural), nationality (Swiss, non-Swiss), educational level (low [compulsory education or less] vs. middle [secondary education] vs. high [tertiary education]), marital status (married/registered partnership vs. single, divorced/dissolved, separated, widowed), smoking status (never, former, current), chronic alcohol consumption associated with health (women ≥20 g, men ≥40 g ethanol daily vs. less), physical activity (≥150 min per week vs. less) [24], paying attention to diet (yes vs. no), and self-perceived health status (fair, poor, very poor vs. very good, good) was assessed.

### Statistical analyses

Analyses were conducted with the Stata statistical software version 13.1 (College Station, TX) and weighted with weighting factors according the Swiss general population. The weights are based on the 2012 Swiss population with respect to sex, age, geographic region and nationality (Swiss/non-Swiss); any differences caused by stratification or non-participation were mathematically corrected. For descriptive statistics, we present means and percentages. Logistic regression analyses examined the associations of BWD with depression (yes/no), and odds ratios (OR) with the corresponding 95 % confidence intervals (CI) were calculated. We computed 4 models successively: 1) unadjusted, 2) adjusted for age and sex, 3) adjusted for confounders chosen a priori, due to the known literature or the expected association with both, BWD and depression, except for BMI, and 4) adjusted for all the covariates in model 3 plus BMI. We used the Wald-test - including the interaction term of BWD with age, sex or BMI - to examine whether age, sex and BMI modulated the association of BWD with depression. *P* < 0.05 was considered to be statistically significant.

## Results

Table 1 shows the selected characteristics of the weighted study population (mean age 47.7 years). Nearly one third (31.3 %) were overweight and 10 % were obese. Normal weight was reported by 56.0 % of individuals and underweight by 3 %. Most participants lived in urban areas (73.4 %), were Swiss (80.8 %), and had a middle educational level (56.5 %). More than half of the participants (53.8 %) were married or lived in a registered partnership. Current smoking was stated by 27.7 % of the study participants and chronic hazardous alcohol consumption by 4.8 %. Not reaching the recommendations for physical activity was reported by approximately one fourth (25.8 %), and not paying attention to diet by 29.8 % of the participants. Self-perceived health was considered as fair, poor or very poor by 14.2 % of the participating individuals. Overall, 6 % of the study participants were above the cut-off point of the PHQ-9, indicating a current major depressive disorder.

**Table 1:** Characteristics of the study sample of the 2012 Swiss Health Survey^a^

		Total
Total, n		15,975
Age, mean (SE)		47.7 (0.18)
Sex	Males	50.2
	Females	49.8
Body mass index (BMI)	Underweight (BMI < 18.5 kg/m^2^)	3.0
	Normal weight (BMI ≥ 18.5 to < 25.0 kg/m^2^)	56.0
	Overweight (BMI ≥ 25 to < 30.0 kg/m^2^)	31.3
	Obesity (BMI ≥ 30 kg/m^2^)	9.7
Area of residence	Urban	73.4
	Rural	26.6
Nationality	Swiss	80.8
	Foreign	19.2
Educational level	High	33.3
	Middle	56.5
	Low	10.2
Marital status	Married/registered partnership	53.8
	Single, divorced/dissolved partnership, separated, widowed	46.2
Smoking status	Never smokers	49.3
	Ex-smoker	23.0
	Current smokers	27.7
Chronic alcohol consumption^b^	No	95.2
	Yes	4.8
Physical activity	≥150 min. per week	74.2
	<150 min. per week	25.8
Pay attention to diet	Yes	70.2
	No	29.8
Self-perceived health	Good, very good	85.8
	Fair, poor, very poor	14.2
Body weight satisfaction	Absolutely satisfied	32.5
	Partly satisfied	43.6
	Rather unsatisfied	19.1
	Absolutely unsatisfied	4.9
Depression	No	94.0
	Yes	6.0

^a^Weighted according to the Swiss general population

^b^Ethanol ≥ 20 g/day for women, ≥ 40 g/day for men vs. less

**Fig. 1:**
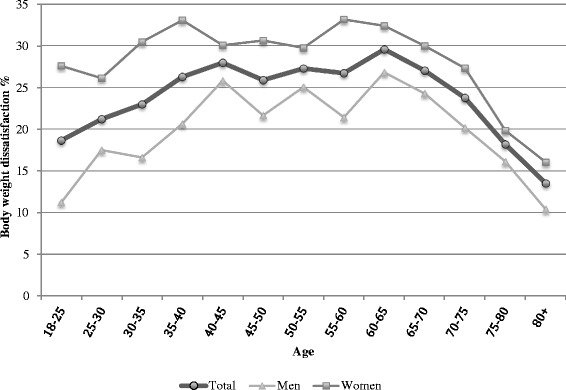
Prevalence of body weight dissatisfaction; 2012 Swiss Health Survey

Barely one third (32.5 %) of the study participants were absolutely satisfied with their body weight, 43.6 % partly, 19.1 % rather unsatisfied and 4.9 % absolutely unsatisfied. As Fig. 1 shows, women across all age groups were more often unsatisfied with their body weight than men. In males and females, the highest peaks of BWD were reached in 35–65 years old.

**Fig. 2:**
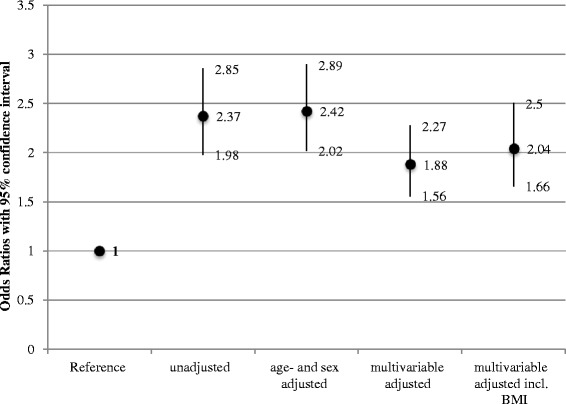
Associations between body weight dissatisfaction and depression assessed with logistic regression analyses and adjusted for different confounders; 2012 Swiss Health Survey. Multivariable adjustment included the variables area of residence, nationality, educational level, smoking status, physical activity, marital status, alcohol consumption, attention to diet, self-reported health, age (if appropriate), sex (if appropriate), and body mass index (if appropriate)

As shown in Fig. 2, BWD was associated with depression (men and women combined OR 2.04, 95 % CI 1.66–2.50; multivariable adjusted). Table 2 presents similar results when looking at the association by sex (OR 1.85, 95 % CI 1.34–2.56 for men, OR 2.25, 95 % CI 1.71–2.96 for women, respectively), independent of the individual’s reported BMI. But results did not differ between men and women (*P*-interaction = 0.13).

**Table 2:** Association between body weight dissatisfaction and depression stratified by sex, age and body mass index; 2012 Swiss Health Survey^a^

	No depression (ref.)	Unadjusted		Age adjusted		Multivariable adjusted^b^		Multivariable adjusted^b^ incl. BMI	
Body weight dissatisfaction	OR	OR	95 % CI	OR	95 % CI	OR	95 % CI	OR	95 % CI
Men	1	1.89	[1.41,2.54]	2.05	[1.54,2.74]	1.60	[1.18,2.18]	1.85	[1.34,2.56]
Women	1	2.76	[2.21,3.45]	2.77	[2.21,3.47]	2.16	[1.72,2.72]	2.25	[1.71,2.96]
*p*-Interaction^c^		0.01		0.09		0.18		0.13	
Sex adjusted									
≥18 to < 30 years	1	1.91	[1.28,2.86]	1.88	[1.27,2.79]	1.53	[1.02,2.30]	1.78	[1.16,2.74]
≥30 to < 60 years	1	2.77	[2.22,3.45]	2.74	[2.20,3.41]	2.05	[1.62,2.59]	2.10	[1.61,2.74]
≥60 years	1	2.63	[1.72,4.02]	2.64	[1.71,4.08]	2.04	[1.29,3.23]	2.34	[1.30,4.23]
*p*-Interaction		<0.01		0.01		0.07		0.08	
Age and sex adjusted									
Underweight (BMI < 18.5 kg/m^2^)	1	5.19	[2.25,11.95]	5.17	[2.14,12.50]	5.20	[1.77,15.26]		
Normal weight (BMI ≥ 18.5 to < 25.0 kg/m^2^)	1	2.52	[1.87,3.41]	2.43	[1.81,3.28]	1.89	[1.39,2.59]		
Overweight (BMI ≥ 25 to < 30.0 kg/m^2^)	1	2.80	[2.02,3.89]	2.46	[1.77,3.42]	2.00	[1.41,2.83]		
Obesity (BMI ≥ 30 kg/m^2^)	1	2.53	[1.41,4.54]	2.19	[1.20,3.98]	1.97	[1.10,3.51]		
*p*-Interaction		0.48		0.49		0.97			

^a^Weighted according to the Swiss general population

^b^Adjusted for area of residence, nationality, educational level, smoking status, physical activity, marital status, alcohol consumption, attention to diet, self-reported health, age (if appropriate), sex (if appropriate), and body mass index (if appropriate)

^c^Interaction term: cross-product of sex, age-groups or weight groups, respectively, with body weight satisfaction

Stratifying by age groups showed significant associations of BWD with depression in young (OR 1.78, 95 % CI 1.16–2.74), middle-aged (OR 2.1, 95 % CI 1.61–2.74) and old individuals (OR 2.34, 95 % CI 1.30–4.23) also independent of BMI. Statistical significance for modification by age groups was not observed (*P*-interaction = 0.08).

By stratifying for BMI category, we found statistically significant positive associations of BWD and depression in underweight (OR 5.2, 95 % CI 1.77–15.26), normal weight (OR 1.89, 95 % CI 1.39–2.59), overweight (OR 2.0, 95 % CI 1.41–2.83), and obese individuals (OR 1.97, 95 % CI 1.10–3.51). We did not observe any statistically significant effect modification by BMI categories (*P*-interaction = 0.97).

## Discussion

Based on data of the 2012 Swiss Health Survey, the present analyses revealed positive associations between BWD and depression independent of sex, age and BMI. To our knowledge, this is the first time these associations are studied in Switzerland.

Depression is the most common psychiatric disease in Switzerland. In the present study, 6 % were affected by a major depressive disorder according to the PHQ-9. Our estimate is in line with the estimated prevalence of 6.9 % in the European Union [25].

BWD has impact on nearly one fourth of the population living in Switzerland. The highest peaks were observed in middle-aged, 35–60 year olds. This proportion is a lot lower than the one found in a cross-sectional population-based German study [6], in which body weight dissatisfaction was reported by 40 % of the 25–74 year old men and women. However, body weight dissatisfaction was not assessed in the same way as in the SHS, which may have resulted in the observed difference.

The observed association between BWD and depression in the present study is in accordance with findings from surveys conducted mainly in adolescents and looking at the associations of various components of body image with depression [2, 19, 26–29]. In a U.S. study [2] on 2,139 adolescent males who were followed into adulthood, boys who had an average weight and perceived themselves as overweight or very underweight stated significantly more depressive symptoms than boys without body weight distortions. This result did not change over the 13-year follow-up period. In another longitudinal study among US adolescents body dissatisfaction was a predictor of depression for females but not for males [28]. In a cross-sectional study among Portuguese adolescents [29] on the other hand, body dissatisfaction contributed to depressive symptoms, without gender differences.

But in general women tend to internalize a thin appearance ideal [30], whereas the ideal male body is one of lean muscularity [2, 31]. Thus, our question on body weight satisfaction may not be precise enough to distinguish between obesity and muscularity. Since both obesity and muscularity dissatisfaction are of importance of the masculine body image upcoming research should assess both of them [2]. Although sex did not modulate the associations between BWD and depression, women tended to be more often dissatisfied than men.

In adults, the evidence is limited and not yet conclusive [5, 8, 16, 17, 32]. A cross-sectional analysis of the American Study of Women’s Health Across the Nation (SWAN) [8] observed that middle-aged women with body image dissatisfaction or who perceived themselves as “unattractive”, but not those with BWD, were more likely to report clinically significant levels of depressive symptoms. Also in a study conducted with 97 mainly female U.S. patients with binge eating disorders, body image disturbance was positively associated with depression [17]. In contrast, a prospective survey among Spanish university graduates, showed no association between body image disturbance and subsequent depression neither in adult men nor in adult women [16].

Others investigated whether body weight categories may modulate the association between BWD and depression. A variety of studies conducted with children or adolescents observed that perception of body weight may be more important than objectively measured weight in the relationship with mental health, e.g. with suicide ideation or attempts [33–36]. Furthermore, in a cross-sectional Chinese Study [19] adolescents who perceived themselves as overweight were more likely to experience depressive symptoms than those who perceived themselves as normal and/or underweight. In the same study, no significant association between depressive symptoms and actual measured weight status was observed. In a population-based cross-sectional American Study (NHANES) with over 13,000 participants, women who perceived themselves as underweight or overweight had an increased odd of depression compared with women who perceived themselves as about the right weight. This association was independent from measured weight. Among men, perceiving oneself as underweight but not being underweight (objectively measured) was associated with depression [32]. Accordingly, in our study, BWD remained to be associated with depression after adjustment for BMI. Thus, we confirmed the results of previous studies showing that perception of body weight might be a better predictor for depression than actual weight status.

Moreover, in our study, BWD was not only positively associated with depression in underweight, overweight and obese individuals but also in those with normal weight. Similarly, a recent cross-sectional study on Chinese adolescents looked at the moderating factors between the association of body dissatisfaction and depression. They observed significant associations in underweight, normal weight and overweight females, but in males associations were only observed in underweight and normal weight adolescents [11].

Potential mechanisms how body dissatisfaction is associated with mental health have been postulated previously [11, 19], focusing on the fact that body dissatisfaction stems from an inappropriate emphasis on the importance of thinness and other unachievable standards of beauty and, thus, may affect depression onset. This hypothesis is supported by the longitudinal study from Stice et al. [13] on female adolescents, in which body dissatisfaction has been identified as a predictor of depression. In this context, experiencing weight stigma predicts poor psychological outcomes including depression, similar to those outcomes who have been linked to higher BMI [37]. On the other hand, an association between body image and depression is also supported by the findings from neurobiological investigations [19]. Deficits in the hypothalamic pituitary-adrenal axis and serotonin system have been shown to be involved in mood disorders as well as in weight regulation. Furthermore, also brain areas which are involved in hedonic regulation may play a role for both body image and depression [38].

Our study has several strengths, including the large, nationally representative sample of individuals 18 years and older living in Switzerland, due to the use of weighting factors, which allows for the extrapolation of the results in relation to age, sex, region and nationality from the sample to the total population. Furthermore, the survey data allowed for adjusting for a number of important covariates associated with BWD and depression, although we were not able to take all potential confounders into consideration, such as family history of depression, medication, and also residual confounding might have occurred. A further strength was that depression was defined using a validated instrument with DSM-IV based criteria. However, our analysis was based on a cross-sectional study, and therefore we cannot exclude reverse causation, i.e. depression may also lead to BWD, which is a major limitation. Nevertheless, as these associations have not been examined yet for Switzerland, it is worthwhile to start with this population-based cross-sectional approach, but longitudinal studies are needed to examine the temporal relationship between body image and depression. Because institutionalized individuals were excluded from the sampling procedure, excluding most probably cases with severe depression, thus the prevalence of depression is possibly underestimated. The fact, that BWD, depression, body weight, height, and potential confounders were based solely on self-report may have biased the results. Finally, it should be noted that although BWD and body image dissatisfaction are related constructs, they are not redundant. The various aspects of body image and depression discussed in the present literature have therefore to be taken into consideration.

## Conclusions

BWD was observed to be widespread and affected nearly one fourth of the population living in Switzerland. In this representative sample, BWD was associated with depression. The association was independent of age, sex, and BMI, i.e. present in individuals with normal body weight and in all age groups. Our results need to be confirmed in longitudinal studies. Presumed that causality can be provided in future longitudinal studies, programs that aim to reduce depression by diminishing body (weight) dissatisfaction should refer to all weight groups.

## Abbreviations

BMI: Body mass index
BWD: Body weight dissatisfaction
CI: Confidence interval
OR: Odds ratio
PHQ-9: Patient health questionnaire, SE, Standard error, SHS, Swiss Health Survey
SFSO: Swiss Federal Statistical Office
